# Sacral Myeloid Sarcoma Manifesting as Radiculopathy in a Pediatric Patient: An Unusual Form of Myeloid Leukemia Relapse

**DOI:** 10.1155/2018/4257012

**Published:** 2018-05-15

**Authors:** Joana Ruivo Rodrigues, Manuel João Brito, Rui Pedro Faria Pais, Sílvia Carvalho

**Affiliations:** ^1^Radiology Unit, Medical Imaging Department, Centro Hospitalar e Universitário de Coimbra, Coimbra, Portugal; ^2^Pediatric Oncology Department, Hospital Pediátrico, Centro Hospitalar e Universitário de Coimbra, Coimbra, Portugal; ^3^Neuroradiology Unit, Medical Imaging Department, Centro Hospitalar e Universitário de Coimbra, Coimbra, Portugal

## Abstract

Myeloid sarcoma (MS), granulocytic sarcoma or chloroma, is defined as a localized extramedullary mass of blasts of granulocytic lineage with or without maturation, occurring outside the bone marrow. MS can be diagnosed concurrently with acute myeloid leukemia (AML) or myelodysplastic syndrome (MDS). The authors report a case of sacral MS occurring as a relapse of myeloid leukemia in a 5-year-old girl who was taken to the emergency department with radiculopathy symptoms.

## 1. Introduction

In 1811, MS was described for the first time by Burns. Afterwards, in 1853, King called the disease chloroma due to its greenish appearance [1]. MS is frequently diagnosed in patients with recurrent AML. Immature myeloid cells or extramedullary proliferation of myeloblasts are present in MS [2]. The incidence of chloroma in AML is approximately 3–9.1% [3].

MS is an uncommon neoplastic condition [4, 5] and any organ system can be affected [1]. The soft tissue of the head, neck, skin, bone, and lymph nodes is usually affected [1]. Spinal MS of the sacrum is infrequent. Compressed spinal cord symptoms caused by an MS mass are unusual [1, 4]. Early diagnosis of MS is possible using enhanced Magnetic Resonance Imaging (MRI). This imaging technique can show the precise location and size of the lesion. Biopsy of the mass is required for a definitive MS diagnosis [4]. MS is a significant cause of morbidity among patients with leukemia.

In this paper, the authors describe the MRI findings and clinical manifestations of an uncommon case of spinal MS at the sacral level in a 5-year-old girl who presented with radiculopathy symptoms and had a previous history of AML.

## 2. Case Presentation

**Figure 1:**
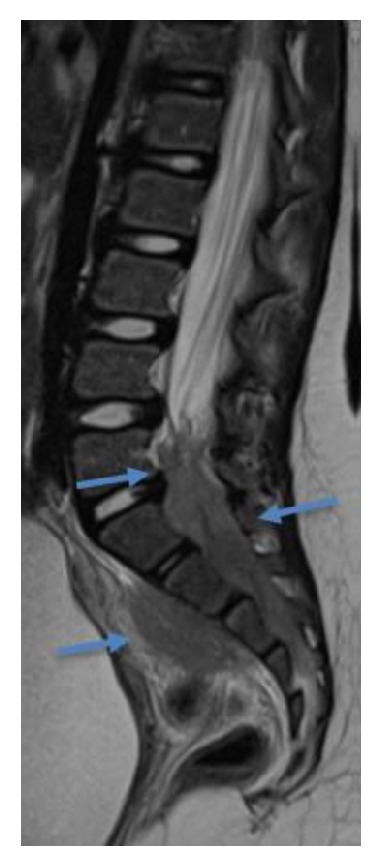
Sagittal T2-weighted MRI of the lumbosacral spine shows a large intracanalar epidural hypointense mass at L5 to S4, extending into the presacral space (blue arrows).

**Figure 2:**
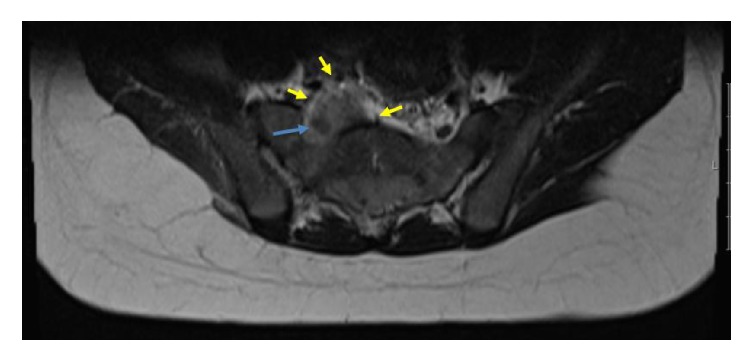
Axial T2-weighted MRI reveals anterior mass extension into the paraspinous soft tissues (yellow arrows), involving the sciatic nerve (blue arrow), which is thickened.

**Figure 3:**
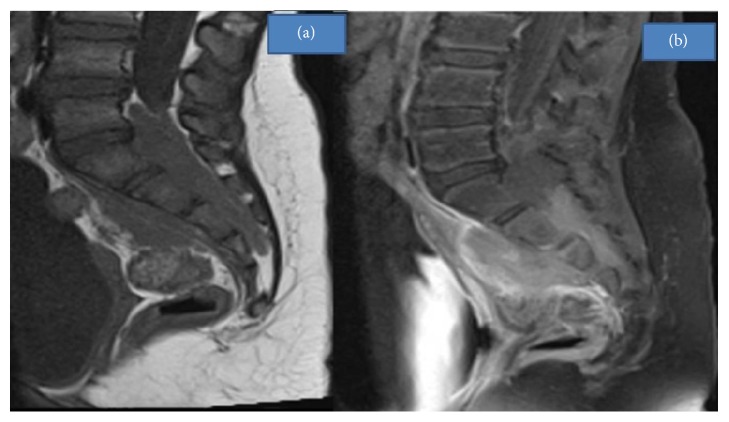
(a) Sagittal T1-weighted MRI shows a hypointense intracanalar and presacral infiltrating mass. (b) Postcontrast T1-weighted sagittal image with fat suppression shows heterogeneous enhancement of the mass and sacral vertebrae.

A 5-year-old girl with acute myeloid leukemia (AML) with t(8;21) underwent relatively standard AML chemotherapy, which consisted of 2 cycles of induction chemotherapy with daunorubicin, cytarabine, and etoposide, followed by 2 cycles of consolidation chemotherapy with high-dose cytarabine 3 gr/m^2^ per dose ×6, in each cycle. Intrathecal therapy was also carried out. The patient was in remission by morphology and flow cytometry assessment after the first cycle and in molecular remission after the second cycle by flow cytometry assessment. Four months after the end of therapy, she presented progressive radicular pain of the lower right extremity and gait claudication. Neurological assessment revealed symmetrical depressed reflexes of the lower limbs and reduction of right knee extension. The posterolateral regions of the right buttock and thigh were painful to the touch. Laboratory evaluation showed normal blood counts. The imaging study evaluation with an MRI of the spine revealed a soft tissue intracanalar epidural mass located from L5 to S4, which extended through multiple sacral neural foramina to the presacral space. The tissue involved sacral nervous roots (Figure 1) and the sciatic nerve (Figure 2). The infiltrating mass was hypointense in T1- and T2-weighted images and enhanced after contrast (Figure 3). Based on the MRI findings, the authors suggested the diagnosis of chloroma. The chest X-ray was normal. In order to confirm the leukemic relapse, a bone marrow aspiration with biopsy and a minimal invasive biopsy of the sacral mass, guided by Computed Tomography, were performed. Bone marrow evaluation showed a hypercellular marrow with 2% myeloid blasts with fluorescent in situ hybridization (FISH) testing and was positive for the t(8;21) ETO/AML1 fusion. Histologic findings of the sacral mass revealed connective tissue without signs of invasion by previously diagnosed neoplasia. Flow cytometry could not verify the etiology of the mass, but ETO/AML1 translocation was confirmed by PCR in the biopsied sacral mass. The 2% of blasts in the bone marrow and the myeloid sarcoma as an epidural mass suggested the diagnosis of relapse combined with AML.

Following the institutional guidelines for relapsed AML, the patient started FLAG-IDA as a relapse protocol. Intrathecal chemotherapy was not performed in the first cycle due to the mass's large volume.

**Figure 4:**
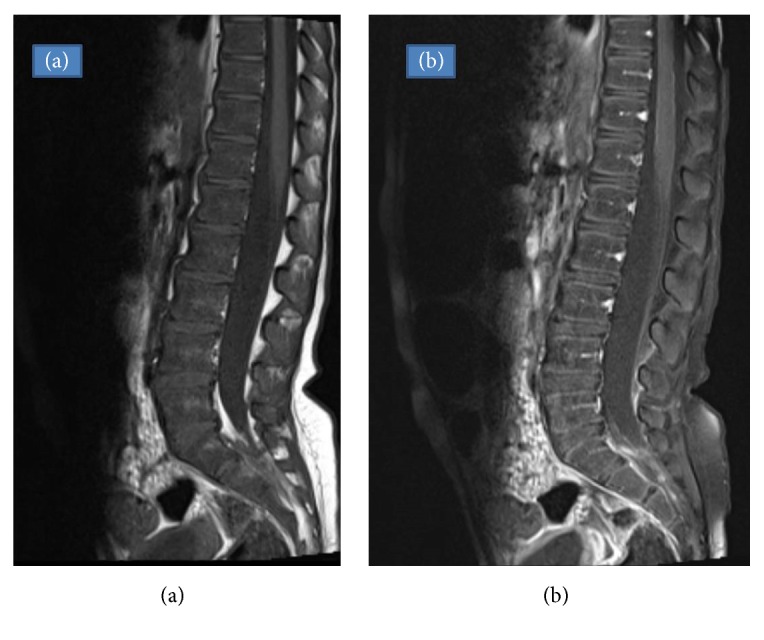
Sagittal T1-weighted image (a) and sagittal postcontrast T1-weighted image with fat suppression (b) show a significant tumor mass volume reduction after treatment.

The patient was in remission by morphology and flow cytometry assessment after the first cycle of FLAG-IDA. Neurological assessment revealed normal and symmetric reflexes of the lower limbs, as well as symmetric and normal knee extension. At that time, the patient did not have pain in the right buttock or thigh. The MRI evaluation revealed a significant tumor mass volume reduction at that time (Figure 4).

Triple intrathecal therapy was performed at the beginning of the second FLAG-IDA cycle and there were no blasts detected in the cerebrospinal fluid. Following the second cycle, the patient experienced a four-month period of hematologic aplasia, which postponed the allogeneic stem-cell transplant (allo-HSCT). Meanwhile, she remained in remission but did not fully recover from the hematologic aplasia, while the platelets levels remained low. She started maintenance chemotherapy, in addition to intrathecal therapy (no blasts in the CNS fluid) as a bridge to allo-HSCT. At this point, the patient is waiting for allo-HSCT. The decision to undergo radiotherapy has been postponed until further evaluation after the transplant, as there was no growth of the sacral mass during the prolonged bone marrow aplasia.

## 3. Discussion

In literature, MS is classified according to the predominant cell type (blastic, monoblastic, and myelomonocytic) [6]. MS can have different clinical presentations. MS can appear simultaneously with AML. The case in this paper presents this type of clinical manifestation. MS can appear with blast phase/transformation of a myeloproliferative neoplasm, also known as chronic myelomonocytic leukemia. MS may also be associated with a normal bone marrow biopsy and blood smear, without a history of myeloid neoplasia [7–9]. Lastly, extramedullary relapse of AML may also occur after a bone marrow transplant procedure [9]. Extramedullary relapse is less frequent in patients treated without allo-HSCT, compared to patients treated with allo-HSCT. Children rarely have extramedullary relapse without bone marrow involvement [10].

Haversian channels serve as a bridge for tumor cell migration from the bone marrow to extramedullary sites. Spinal cord involvement is infrequent [11] and the spine segments most often affected are the thoracic followed by the lumbar, sacral, and cervical segments [12]. 18% of patients have multiple noncontiguous areas of involvement [13]. Lesions that have intraspinal invasion [14], as in the case described, may cause cord compression or* cauda equina syndrome*. Those complications are rare [13].

AML reservoirs are usually bone marrow, the central nervous system, and the testes. The sacral spinal canal is a specialized AML reservoir [15] and the average age for sacral MS manifestation is 22 years, being more predominant in males than in females [13]. The risk factors for extramedullary relapse are advanced disease conditions at the time of HSCT, chromosomes 5q and 7q deletions and FLT-3 mutations, history of an extramedullary disease, and FAB classification M4 or M5 AML [10].

Langerhans cell histiocytosis X, lymphoma, metastatic tumors, and Ewing sarcoma/PNET are the differential diagnoses of MS [16]. Bone marrow aspiration, biopsy, peripheral blood smears, and immunohistochemical detection are used to diagnose MS. The most suitable marker to identify MS is staining for myeloperoxidase. MS masses express lysozyme, Ki67, and myeloid/monocytoid antigens (CD13, CD14, CD33, CD64, CD68, and c-Kit (CD117)) [4].

MRI is the best imaging study to distinguish MS from other pathologies [4]. During MRI, MS commonly appears as a heterogeneous signal mass with isosignal or hypersignal intensity in T2-weighted images and with hyposignal or isosignal intensity in T1-weighted images, when compared to brain or muscle tissue [17]. After intravascular contrast, the MS masses usually present homogeneous enhancement [4]. In the case presented by the authors, the lesion was hypointense in T2-weighted images and hypointense in T1-weighted images and showed heterogeneous enhancement in postcontrast MRI images. The outcome of patients with systemic relapse is worse in comparison to the outcome of patients with isolated extramedullary relapse [10]. This is what occurred in this specific case.

Systemic chemotherapy using AML-like regimens should be initiated early, even in nonleukemic disease [9], with intrathecal chemotherapy as another option [13]. When the lesions are symptomatic or cause local organ dysfunction, surgery or/and radiotherapy might be carried out [9, 13]. In patients with complete remission, allo-HSCT can be performed [9]. There are no established treatment strategies for MS [4, 10]. None of the different specific treatments increase survival [13].

In this paper, the authors describe a rare case of leukemia relapse, presented as sacral MS in a girl with a progressive right-sided lumbosacral plexus neuropathy. The reader should bear in mind that sacral MS presents symptoms similar to other spinal lesions. MS should not be neglected and should be maintained under the differential diagnosis of spinal mass lesions. MRI, bone marrow aspiration, and pathological and histological staining can result in its correct diagnosis.

## Data Availability

The data used to support the findings of this study are available from the corresponding author upon request.

## References

[1] Seok J. H., Park J., Kim S. K., Choi J. E., Kim C.-C. (2010). Granulocytic sarcoma of the spine: MRI and clinical review.

[2] Oliveira E., Lavrador J., Teixeira J., Pignatelli A., Livraghi S. (2016). A purely extradural lumbar nerve root cavernoma mimicking acute myeloid leukemia recurrence: case report and literature review.

[3] Ferri E., Minotto C., Ianniello F., Cavaleri S., Armato E., Capuzzo P. (2005). Maxillo-ethmoidal chloroma in acute myeloid leukaemia: case report.

[4] Lama S., Lui S., Xiao Y., Zhang H., Karki M., Gong Q. (2015). Sacral myeloid sarcoma involving multiple metastases to the brain: A case report.

[5] Valsamis E. M., Glover T. E. (2017). Granulocytic sarcoma: A rare cause of sciatica.

[6] Pileri S. A., Ascani S., Cox M. C. (2007). Myeloid sarcoma: clinico-pathologic, phenotypic and cytogenetic analysis of 92 adult patients.

[7] Siraj F., Kaur M., Dalal V., Khanna A., Khan A. A. (2017). Myeloid sarcoma: a report of four cases at unusual sites.

[8] Orofino N., Cattaneo D., Bucelli C. (2017). An unusual type of myeloid sarcoma localization following myelofibrosis: A case report and literature review.

[9] Almond L. M., Charalampakis M., Ford S. J., Gourevitch D., Desai A. (2017). Myeloid sarcoma: presentation, diagnosis, and treatment.

[10] Samborska M., Derwich K., Skalska-Sadowska J., Kurzawa P., Wachowiak J. (2016). Myeloid sarcoma in children—diagnostic and therapeutic difficulties.

[11] Mostafavi H., Lennarson P. J., Traynelis V. C. (2000). Granulocytic sarcoma of the spine.

[12] Landis D. M., Aboulafia D. M. (2003). Granulocytic sarcoma: An unusual complication of aleukemic myeloid leukemia causing spinal cord compression. A case report and literature review.

[13] Siker M. L., Bovi J., Alexander B. (2016). Spinal cord tumors.

[14] Dhakar M. B., Bansal P., Tselis A. C. (2015). Sacral spine myeloid sarcoma.

[15] McCarty S. M., Kuo D. J. (2017). Persistent sacral chloroma in refractory acute myelogenous leukaemia.

[16] Inoue T., Takahashi T., Shimizu H. (2008). Spinal granulocytic sarcoma manifesting as radiculopathy in a nonleukemic patient.

[17] Pui M. H., Fletcher B. D., Langston J. W. (1994). Granulocytic sarcoma in childhood leukemia: imaging features.

